# ECG-Based Identification of Sudden Cardiac Death through Sparse Representations

**DOI:** 10.3390/s21227666

**Published:** 2021-11-18

**Authors:** Josue R. Velázquez-González, Hayde Peregrina-Barreto, Jose J. Rangel-Magdaleno, Juan M. Ramirez-Cortes, Juan P. Amezquita-Sanchez

**Affiliations:** 1 Department of Computational Science, National Institute of Astrophysics, Optics, and Electronics, Santa Maria Tonantzintla, Puebla 72840, Mexico; josueg@inaoep.mx; 2 Department of Electronics, National Institute of Astrophysics, Optics, and Electronics, Santa Maria Tonantzintla, Puebla 72840, Mexico; jrangel@inaoep.mx (J.J.R.-M.); jmram@inaoep.mx (J.M.R.-C.); 3 Facultad de Ingeniería, Universidad Autónoma de Querétaro, Av Río Moctezuma 249, San Juan del Rio 76807, Mexico; jamezquita@uaq.mx

**Keywords:** ECG signals, sparse representations, sudden cardiac death

## Abstract

Sudden Cardiac Death (SCD) is an unexpected sudden death due to a loss of heart function and represents more than 50% of the deaths from cardiovascular diseases. Since cardiovascular problems change the features in the electrical signal of the heart, if significant changes are found with respect to a reference signal (healthy), then it is possible to indicate in advance a possible SCD occurrence. This work proposes SCD identification using Electrocardiogram (ECG) signals and a sparse representation technique. Moreover, the use of fixed feature ranking is avoided by considering a dictionary as a flexible set of features where each sparse representation could be seen as a dynamic feature extraction process. In this way, the involved features may differ within the dictionary’s margin of similarity, which is better-suited to the large number of variations that an ECG signal contains. The experiments were carried out using the ECG signals from the MIT/BIH-SCDH and the MIT/BIH-NSR databases. The results show that it is possible to achieve a detection 30 min before the SCD event occurs, reaching an an accuracy of 95.3% under the common scheme, and 80.5% under the proposed multi-class scheme, thus being suitable for detecting a SCD episode in advance.

## 1. Introduction

Sudden cardiac death (SCD) is an unexpected death caused by cardiovascular problems [1] with or without a history of heart disease [2,3]. In general, SCD occurs within an hour after the onset of symptoms, although the person has no history of a fatal heart condition [4]. SCD accounts for more than 50% of all deaths from cardiovascular disease [1], ranking second as the leading cause of death, after cancer [5]. SCD is a vital challenge for clinicians, as it can be experienced in individuals with no history of heart diseases. Numerous heart diseases lead to SCD, such as ventricular tachyarrhythmias (VTA), ventricular tachycardia (VT), ventricular fibrillation (VF), bradyarrhythmia (BA), coronary artery diseases (CAD), valvular diseases (RV), myocardial infarction (MI) and genetic factors [6]. However, deaths by SCD are related to ventricular tachyarrhythmias (including VF and VT) and BA [7], making the heart unable to pump blood effectively. The VF is an underlying quality in most SCD episodes and is considered the leading cause and possible detonator [8,9,10], representing about 20% of SCD episodes. The survival rate decreases approximately 10% per minute for patients after VF onset [1]. Therefore, an early prediction of SCD in a person suffering a VF is of great value for timely intervention, increasing the survival rate.

Predicting an SCD is vital since several actions can be taken to counteract it. For example, the Public Access Defibrillation (PAD) procedure rescues patients from impending death after collapse. However, the success rate of cardiac function restoration primarily depends on when first aid is given to stimulate the heart [11]. It would be preferable to prevent the onset of SCD by providing medical aid before the collapse occurred, which leads to the question of whether it would be possible to have warning systems capable of recognizing cardiac arrest half an hour before the crisis [12]. Efforts have been made regarding this severe health problem, developing efficient ways of predicting the SCD through invasive and non-invasive techniques [13,14,15]. The main goal is to predict the SCD before its onset using ECG signals [13,16], since ECG is one of the most important physiological signals to identify cardiac abnormality and electrical conductivity features. Recent works have experimented with features of ECG and heart rate variability (HRV), a signal extracted from ECG, to detect the subtle changes that occur within the signals before an SCD and to identify in advance a possible SCD risk. Also, additional features (time, frequency, time-frequency, and non-linear) and machine learning algorithms have been used to predict SCD from ECG and HRV signals. According to recent reports, an SCD could be predicted up to 25 min before its onset through intelligent signal processing methods [17,18]. Thus, tools such as diagnostic support systems based on computational analysis and signal processing techniques have been shown to help detect SCD in advance.

In [19,20], an automated prediction of SCD based on HRV signals was performed. Signals were analyzed through techniques that identify data repeatability or time-frequency features such as the Recurrence Quantification Analysis (RQA) and the Discrete Wavelet Transform (DWT); statistics features such as entropy also were used. One important issue in this works is that data analysis can generate a large set of features. Then, a feature reduction is required to consider only the more relevant features; for this purpose, some analysis such as Kolmogorov complexity or feature ranking, commonly based on the t-test, are used. To reduce the information that the classifier has to process is an advantage of these works. In both cases, a prediction up to 4 min before SCD was reached through k-Nearest Neighbor (kNN) and SVM (Support Vector Machine) classifiers having an accuracy of 86.8% and 94.7%, respectively. Prediction time was increased up to five minutes with an accuracy of 93.71% when the kNN classifier processed time-domain features extracted from HRV signals [21]. One disadvantage of SCD detection based on HRV signals is that computational time increases [17], which could be an issue to consider in an application where time is relevant. Therefore, SCD detection also has been studied by directly using ECG signals. In [16], the authors used a simplified evaluation of ECG signals based on a proposed Sudden Cardiac Death Index (SCDI) for the prediction of SCD. The SCDI integrates a weighted combination of the main features identified in the ECG signal and provides a way to obtain a unique value, which is able to differentiate between normal and SCD classes. The classification with SCDI and SVM reached 98.68% accuracy up to four minutes before SCD. A different prediction approach was proposed in [22], where the authors analyze how ECG signal features change in consecutive time intervals. With this analysis, the time resolution of the prediction process was increased, and, using a multi-layer perceptron (MLP) classifier, it was possible to predict SCD 12 min before onset. Recently, an approach to SCD prediction based on ECG signals was presented in [18]. This approach employs the Wavelet Packet Transform (WPT), which considers high-frequency bands in the ECG decomposition, reaching an accuracy of 95.8% and a prediction 20 min before onset. However, the frequency bands are fixed and depend on the sampling frequency, inhibiting the analysis of frequencies defined by the user. An alternative was to use Empirical Mode Decomposition (EMD), a technique able to separate the ECG signal into a set of frequency bands based on its information. In this way, a prediction 25 min before SCD was possible, with 94% accuracy [17].

However, these predictions were made by considering a binary classification in normal and SCD signals. The main drawback of this comparison scheme is that the ECG signal of a patient could contain features that differ from a normal ECG signal due to, for instance, previous heart disease, but not necessarily because of a future SCD episode. Therefore, this evaluation could not be accurate since there is a high probability of SCD misdetection.

This work addresses the feature change in the ECG signal that occurs as the SCD event becomes closer, since this could help in early identification. A methodology based on sparse representations allows distinctive features to be found in normal and previous SCD signals. If these ECG signals are analyzed at different intervals before SCD, and their features are learned, a likely SCD episode could be identified in advance. The learned dictionaries allow a dynamic feature representation of the signal to be obtained, providing a certain flexibility degree to recognize the intraclass variation and improve the description and identification of SCD signals. Moreover, this approach considers a novel multi-class scheme that makes it possible to distinguish a previous SCD signal from a normal signal and, additionally, to more accurately know if this related to a closer or further time interval from the SCD.

Following this, Section 2 contains the proposed method. The experiments designed to evaluate the feasibility of the proposed method, and the results achieved, are described in Section 3. Finally, conclusions and future work are indicated in Section 4.

## 2. Materials and Methods

**Figure 1:**
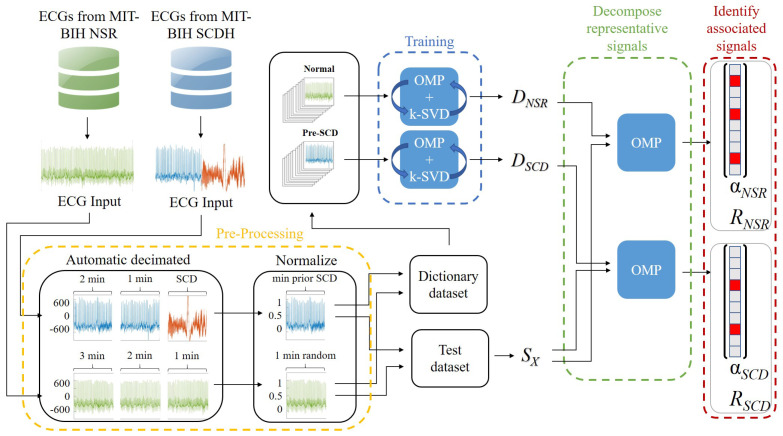
Framework for ECG signal analysis: pre-processing step to obtain 1 min intervals from normal and SCD signals (yellow block), a training step for recognizing particular features from the intervals of interest (blue block), and identification of test signals through their decomposition by sparse representations (red block). In this approach, vector α is considered the feature vector.

**Figure 2:**
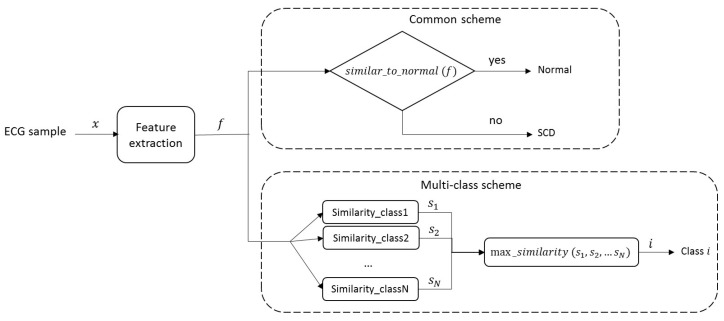
Proposed multi-class scheme and its comparison with the common scheme for SCD signal classification.

A block diagram of the proposed methodology is presented in Figure 1. As a first step, an automatic decimated as a function of time (*t*) is applied to segment the ECG signal; then, the ECG signals are normalized. The generation of the signal basis (dictionaries) is performed in the training phase through the OMP and k-SVD algorithms. The training enables dictionaries to learn the main features of each signal set. Thus, it is expected that the dictionaries help to recognize similarities with a test signal through its decomposition. The common scheme for classifying pre-SCD signals consists of comparing the features of an input signal *x* with the features of two sets of signals, normal and pre-SCD. If the features of *x* are not similar to the normal signals, then *x* corresponds to an SCD signal. This evaluation may generate bias in classification, since any signal that differs from normal signals will be associated with an SCD class. A modification to the common scheme is proposed by using a multi-class evaluation of the signal. In this scheme, several classes are considered; for instance, the normal class and some time intervals pre-SCD. Then, *x* will be associated with the class of higher similitude. The difference between the two schemes is illustrated in Figure 2.

### 2.1. Dataset

**Figure 3:**
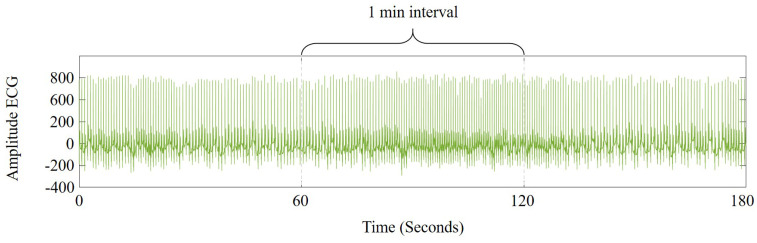
Example of an ECG signal from a healthy subject.

**Figure 4:**
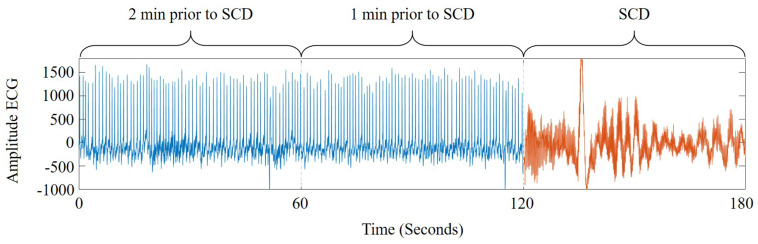
Example of an ECG signal from a subject that suffered an SCD episode.

The data obtained from two international open access databases were used to evaluate the proposed methodology. The ECG signals (normal and SCD) were obtained from the MIT/BIH Normal Sinus Rhythm (NSR) [23] and the MIT/BIH Sudden Cardiac Death Holter (SCDH) [24] databases. In the case of the NSR database, ECG signals of 18 patients are included. Experts from the Arrhythmia Laboratory, at Boston’s Beth Israel Hospital, confirmed that signals belong to subjects with a healthy heart rate, as shown in Figure 3. On the other hand, the SCDH database includes the ECG signals of 23 subjects with SCD caused by VF; these signals were obtained from the Boston area hospitals. Each signal contains a recording of 24 h, including the exact time of the SCD. Three recordings were excluded because they presented heart alterations that differed from an SCD or VF episode. Figure 4 shows an ECG signal from a patient 2 min before the SCD occurrence. Table 1 summarizes some patient features. The clinical information of patients and the time of SCD onset are registered in the SCDH database [24].

### 2.2. Pre-Procesing

**Figure 5:**
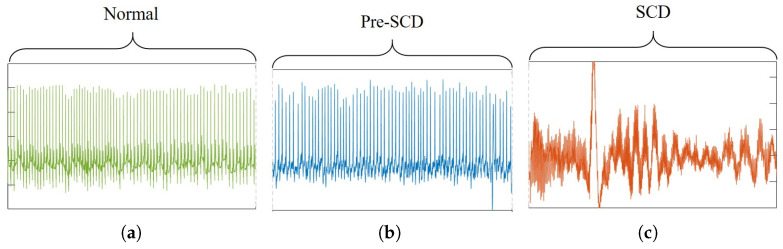
Comparison of segments from ECG signals: (**a**) normal, (**b**) pre-SCD, and (**c**) during SCD.

**Figure 6:**
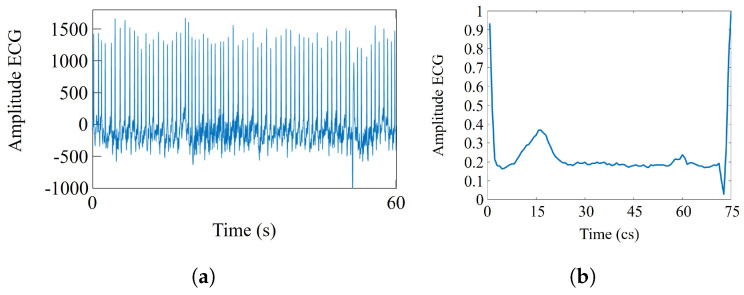
(**a**) 1-min interval from a pre-SCD signal and (**b**) an R-R interval extracted from it.

The ECG signals of the NSR and SCDH databases were acquired at 128 Hz and 250 Hz sampling frequencies, respectively, and digitized with an analog-to-digital converter of 12 bits [24]. To perform the analysis between SCD and control groups, the ECG signals of the SCD group were downsampled to 128 Hz by convolving the signal with a low-pass Finite Impulse Response (FIR) filter. Significant SCD symptoms generally occur within one hour before onset (pre-SCD signals), even though the person does not have a history of fatal heart condition [4]. Since the pre-SCD signals have significant features that can be associated with an SCD event (see Figure 5), they are used for prediction tasks. Then, during analysis of pre-SCD signals, the goal is to detect significant changes that allow for the prediciton of SCD using time intervals of 1 min [16,19,20,21]. In this work, the minutes 5, 10, 15, 20, 25, and 30 before the SCD were analyzed. Additionally, the 1 min interval of the control group is randomly extracted from the ECG signal. All the segments, pre-SCD and control, were normalized, and their respective R-R intervals were extracted, as shown in Figure 6. In a 1 min interval, there are about 70 R-R intervals, since an R-R interval lasts approximately one second; see Figure 6b. Then, there are about 1260 samples for the normal interval (18 subjects) and 1400 samples for each pre-SCD interval (20 subjects). These samples were put into sets corresponding to each of the classes considered in this work, i.e., *C* = {NSR, 5 min, 10 min, 15 min, 20 min, 25 min, 30 min}. Finally, the samples were analyzed to find their particular features and classify them.

### 2.3. Sparse Signals

**Figure 7:**
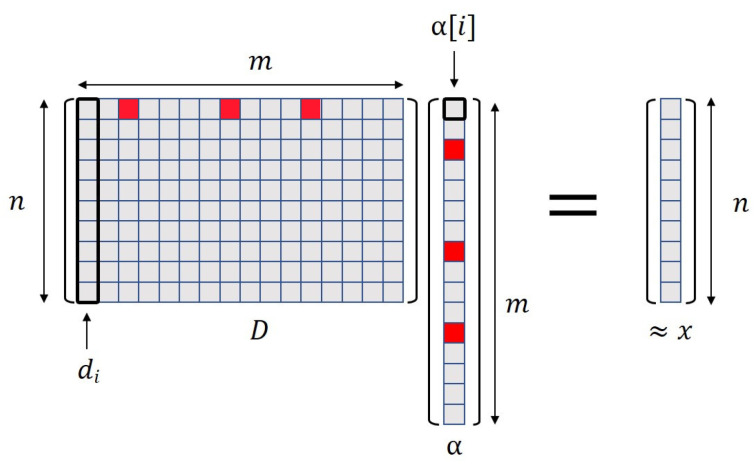
Reconstruction (synthesis) process of a signal *x* using its sparse representation α and a dictionary *D*. Coefficients in α are related to the atoms or elemental signals in *D*; therefore, an approximation of the original signal ≈x can be obtained.

A signal $x_{n\times 1}$, considered as a vector in a finite-dimensional subspace $R^{n}$, is strictly or exactly sparse if most of its entries are equal to zero, i.e., if the set of values F(x)={1≤i≤n∣x[i]≠0} is of cardinality y≪n. The signal *x* can be modeled as the linear combination of *m* elemental signals (atoms), such that

(1) $$\begin{array}{c} x\approx D\alpha =\sum_{i=1}^{m}\alpha \left[i\right]d_{i} \end{array}$$

where $\alpha_{m\times 1}$ is the sparse representation of *x* containing the coefficients associated with the atoms ($d_{i}$) in a matrix dictionary $D_{n\times m}$ involved in the decomposition [25,26] (see Figure 7). Signals sparsed by *D* are written as a superposition of a small fraction of the atoms in the basis. An atom $d_{i}$ of n×1 is an elemental signal representing part of the energy or featuring a specific type of signal to which the dictionary was adapted. Thus, a dictionary *D* is an indexed collection of *m* atoms, i.e., a n×m matrix, whose columns are the atoms. When the dictionary has more columns than rows, m>n, is called overcomplete or redundant, and has a setting in which x≈Dα. Two possible operations can be performed on a dictionary: analysis and synthesis. The analysis is the operation that obtains the sparse representation α of a complete signal *x* by using the expression $\alpha =D^{\prime }x$, where $D^{\prime }$ is the transpose dictionary. The synthesis performs an approximate reconstruction of *x* using Equation (1), as shown in Figure 7.

In previous works, overcomplete dictionaries have demonstrated a high performance in classification tasks [27,28]. There are two types of dictionary: a fixed dictionary and a learned dictionary. Fixed dictionaries contain predefined signals, usually generated by a known function, e.g., sine or wavelets, and provide an analysis operation in a reasonable processing time. When the signals to be analyzed have well-identified features, a fixed dictionary is the best option; otherwise, a learned dictionary must be created. A learned dictionary implies a learning process in which the particular features of a signal set must be captured and recognized through an analysis process. Although dictionary learning means a higher processing time, this option achieves a better performance when the fixed existing dictionaries do not accurately represent the signals that need to be processed [27,28,29].

**Figure 8:**
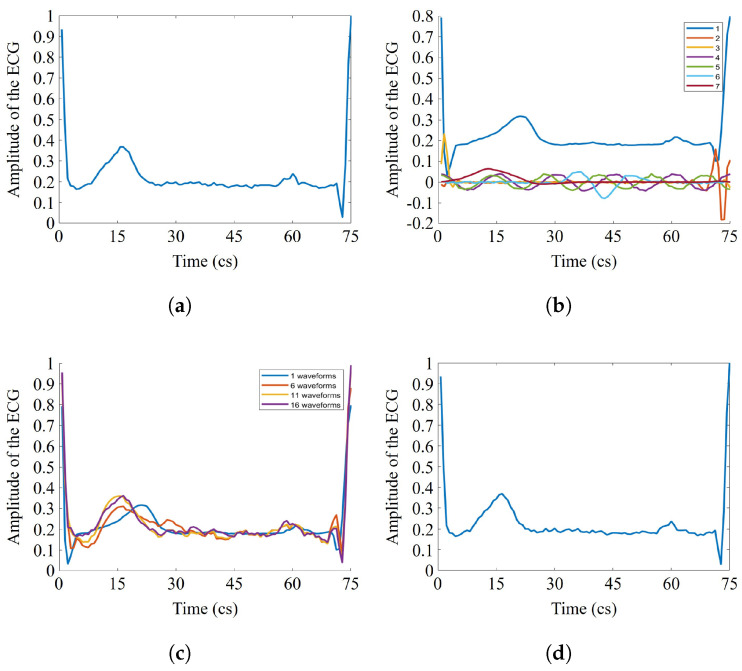
(**a**) Original signal, (**b**) signal decomposition (analysis) in elementary waveforms, (**c**) signal reconstruction (synthesis) by using different number of atoms, and (**d**) signal reconstruction with all numbers of atoms.

Once a dictionary has been defined, it is possible to obtain a sparse representation of the signals. For instance, Figure 8a shows an R-R interval of the original ECG signal. This signal has been decomposed in atoms, along with their corresponding coefficients, through the analysis operation. The atom $d_{i}$ and its α[i] coefficient generate an elemental waveform that represents a part of the original signal (see Equation (1)). Figure 8b shows seven of the sixteen elemental signals in which the original signal was decomposed, while Figure 8c shows its reconstruction through the synthesis operation by using a different number of waveforms. The higher the number of signals used in the reconstruction, the more similar characteristics of the reconstructed and the original signal (Figure 8d).

### 2.4. Dictionary Learning

A trained dictionary is obtained through a dictionary-learning process. In this work, the dictionary-learning process is performed by two algorithms: Orthogonal Matching Pursuit (OMP) and k-Singular Value Decomposition (k-SVD). OMP, a greedy algorithm, reduces the resource requirements and obtains a sparse solution by performing the analysis operation, given a dictionary [25]. After this, k-SVD evaluates how accurate the dictionary is for decomposing the input signals. Both algorithms and their use in SCD prediction are explained in detail in this section.

#### 2.4.1. Orthogonal Matching Pursuit (OMP)

The OMP algorithm searches for an approximate solution through the selection and combination of atoms in *D* that minimize the error-constrained (Equation (2)) sparse coding problem, where ${∥\alpha ∥}_{2}=\sqrt{\sum_{i}{\left|\alpha_{i}\right|}^{2}}$ is the $\ell_{2}$ norm, and ε is the error threshold in the range [0,1]. Thus, Equation (2) allows for signal decomposition until an ε error level is reached; therefore, the number of coefficients may vary from one signal to another.

(2) $$\begin{array}{c} \alpha =\underset{\alpha }{\arg\min}{∥\alpha ∥}_{0}\;\;\;s.\;t.\;\;\;{∥x-D\alpha ∥}_{2}^{2}\leq \varepsilon \end{array}$$

The OMP error-constrained is described in Algorithm 1, where the inputs are the dictionary *D*, a signal *x*, and a given minimum error ε; the expected outputs are the α vector and a residual $r_{j}$. The algorithm ensures that e<ε and $r_{0}=x$, since the signal *x* was not yet decomposed. *I* is the vector of dimensions j×1 that stores the indexes of atoms involved in the decomposition of *x*. The OMP algorithm performs an iterative process that chooses the optimal local solution from a set of possible solutions. In each iteration *j*, this process tries to find, in *D*, the atom $d_{i}$ with the highest correlation to the current energy of the residual $r_{j-1}$ (Algorithm 1, lines 3–4). The index of the *i*-th atom fulfilling the argmax condition is stored in *I* at each iteration, helping to compile the submatrix $D_{I}$. The α vector is computed with the atoms in $D_{I}$; the residual is updated as $r_{j}$, containing the remaining energy of *x*, which is not yet represented by $D_{I}\alpha$ (Algorithm 1, lines 5–6). Finally, *e* is estimated by the ratio between the energy remaining in $r_{j}$ and the energy of the original signal (Algorithm 1, line 7). Thus, OMP generates a set of local optimal solutions, allowing it to find the optimal global solution for the sparse representation α.
**Algorithm 1:** Orthogonal Matching Pursuit.
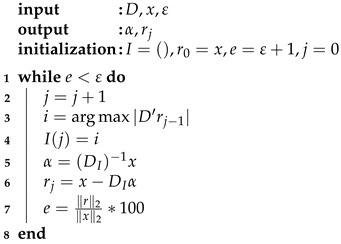


#### 2.4.2. k-SVD

As mentioned above, a dictionary can be adapted to recognize the characteristics of a specific type of signal. k-SVD is an algorithm that allows for the learning process to provide a basis according to a set of signals. Therefore, this is called dictionary learning (Algorithm 2). The process starts from a set *X* containing *M* training signals of the same type, an initial dictionary $D_{0}$, and a given number *K* of iterations; according to the literature, between 10 and 20 iterations are required [30,31]. The end goal is to capture the essential characteristics of the signal set in a final learned dictionary $D_{K}$. First, the matrix of sparse representations, $\alpha_{k}$ of dimensions m×M, is obtained by using OMP and the dictionary $D_{k-1}$ (Algorithm 2, line 2). Since OMP has analyzed a set of signals of the same type, the $\alpha_{k}$ matrix should contain some common atoms in the decomposition of the signals. It is assumed that if an atom takes part in the decomposition of several signals, it adequately represents part of the energy of the signals in *X* and must be preserved; otherwise, it must be recomputed. Then, the *m* atoms of $D_{k-1}$ are analyzed to find their participation in $\alpha_{k}$. For this, the set of signals *w* in which the *j*-th atom takes part (Algorithm 2, line 4) is obtained. A submatrix $\alpha_{w}$ is generated by containing the *w* columns in $\alpha_{k}$ and setting its *j* row to 0, with the aim of performing a signal reconstruction without the participation of the *j*-th atom. Then, the residual matrix (*R*) is computed by the difference in values between the original signal set *X* and the product of the current dictionary with the current coefficients (Algorithm 2, lines 5–6). In this way, the residual *R* between the subset of original signals $X_{w}$ and their reconstruction $D_{k}\alpha_{w}$ provides a more accurate approximation of the *j*-th atom when it is processed by SVD (Algorithm 2, line 7); where *U* are the eigenvalues, *V* the eigenvectors and Σ the diagonal matrix containing the singular values in descending order. The update of the *j*-th atom and its respective coefficient, $d_{j}$ and $\alpha_{j}$, is computed in lines 8–9 of Algorithm 2. It is expected that, in the first iterations, $D_{k-1}$ does not provide an accurate decomposition $\alpha_{k}$ but that the dictionary’s ability to represent *X* improves as *k* increases, until it reaches $D_{K}$.
**Algorithm 2:** K-Singular Value Decomposition.
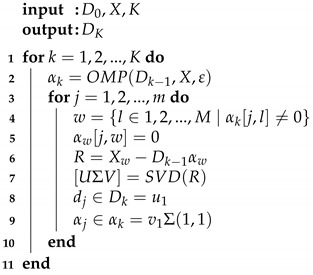


## 3. Results and Discussion

In ECG applications, it is expected that, by using α as a feature vector, the sparse representation helps to distinguish between the different ECG signals (normal and SCD). Since the aim is the early detection of changes in an ECG signal, which could be associated with a possible SCD, two general steps were followed in this methodology: (i) dictionary learning, to identify the features of each signal class in *C* and (ii) signal classification, by measuring the similarity between the features of a new input signal and the learned features for each class.

To identify the features of the signals considered in *C*, a trained dictionary is necessary for each class. Through the learning process with k-SVD, a dictionary identifies the common elemental signals of a particular class. As mentioned in Section 2.2, *C* considers the samples for normal ECG signals and six time intervals at 5 min, 10 min, 15 min, 20 min, 25 min, and 30 min previous SCD, i.e., seven classes in total. Therefore, seven trained dictionaries are required to perform ECG signal classification based on sparse representations. For dictionary learning, it is necessary to have a set of samples of the same type from which the common elemental signals can be identified through the k-SVD algorithm. For this purpose, the samples in each class of *C* were randomly selected and divided into test and training subsets; the division of the training and test sets followed a 55–45% relationship, i.e., for the training and test sets, ten and eight recordings were taken from the MIT/BIH NSR database, and eleven and nine recordings from the MIT/BIH SCDH database. No recording from the training stage was used for the test stage. Thus, the k-SVD was performed, receiving an initial dictionary $D_{0}$ filled with random values, the training set of samples of a particular class c∈C, and K=20 iterations as parameters. The maximum number of iterations was set to ensure the dictionary was completely trained; fewer iterations could reduce the performance during signal decomposition. As a result, the dictionary $D_{c}$, which was specifically adapted to recognize the elemental signals of class *c*, is obtained. This process is repeated for all the classes of interest; in this case, for all the classes in *C*. Therefore, a set of trained dictionaries $DT=\{D_{NSR},D_{5\min},D_{10\min},D_{15\min},D_{20\min}$, $D_{25\min},D_{30\min}\}$ are used to obtain the most accurate decomposition of their respective signals, which can be used for signal classification.

The class of a new input signal *x* must be identified based on the information that is contained in dictionaries. For this, it is necessary to obtain a description of the signal through its features. The α vector obtained by the sparse representation simplifies the signal that can be used as a feature vector. Due to the dictionary’s training process, where more relevant elemental signals were selected, a feature-ranking process is not necessary. The α vector corresponding to *x* must be evaluated to find the higher similitude between its features and the features of a specific set of signals, i.e., a dictionary in DT. To perform features evaluation, it is necessary to obtain α and, to find the higher similitude, *x* must be sparse by all the dictionaries in DT. The OMP algorithm is used to sparse *x* (Algorithm 1), with the learned dictionary for a class $D_{c}\in DT$, the input signal to classify *x* and an error value ε=0.05 as parameters; a high value of ε limits the level of signal decomposition. This process is repeated for each class; then, a set of feature vectors $\alpha_{C}$ is obtained. For classification, it is assumed that a dictionary with learned features of a particular class must recognize a signal of the same type more easily than other dictionaries, as reported in [32]. One way of measuring the recognition of the signal that each dictionary performs is by assessing α coefficients. For instance, if $x_{c1}$ is a signal of class c1, then the dictionary $D_{c1}$ will be able to represent the signal without generating high coefficients, because most of the $x_{c1}$ features are already contained in the elemental signals of $D_{c1}$. Thus, $\alpha_{C}$ can be evaluated by the minimum sum of coefficients, as indicated in Equation (3), and the class of the *i*-th dictionary is the most likely class to be associated with signal *x*. Moreover, having a trained dictionary composed of elemental signals that participate in the ECG decomposition without a specific ranking allows for a dynamic feature extraction process. For example, two samples belonging to the same class could be decomposed by combining different elemental signals from the dictionary. Their energy will be well-represented in their α vectors, since all the elemental signals in the dictionary were adapted for the same type of signal. In this way, a certain flexibility is reached in the feature selection, avoiding the use of both a fixed number of features and a fixed ranking.

(3) $$\begin{array}{c} i=\arg\min_{C}\sum \left|\alpha_{C}\left[i\right]\right| \end{array}$$

For the classification stage, two experiments were performed under the common scheme and the multi-class scheme. To guarantee that signal selection in a classification experiment does not affect the final results, a two-fold cross-validation was computed, then repeated ten times. The obtained results under the proposed methodology were evaluated by using the accuracy (Acc) measure as presented in Equation (4), where true positives (TP), true negatives (TN), false negatives (FN), and false positives (FP) were considered. Moreover, the results were also compared with those obtained in the related works.

Accuracy (Acc): the ratio of correct predictions to the total predictions.

(4) $$\begin{array}{c} Acc=\frac{TP+TN}{TP+TN+FN+FP} \end{array}$$

**Figure 9:**
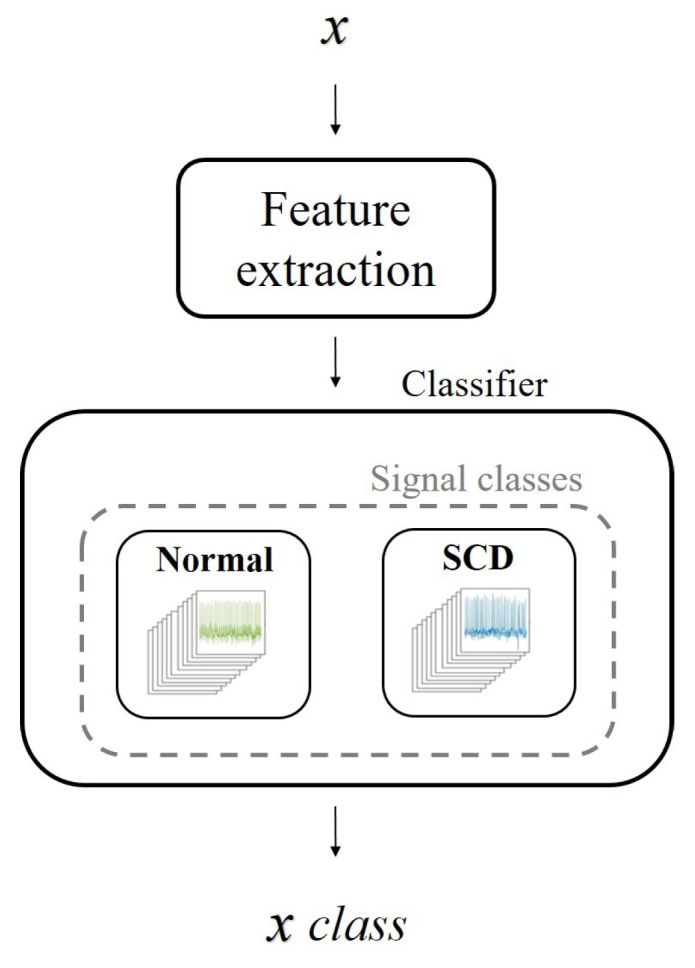
The common scheme used in SCD ECG signal classification, where an input ECG signal is identified as normal or SCD depending on its features; if a signal does not fit the characteristics of one class, then it is assumed to belong to the other.

The sparse representations of processed signals were tested under the common scheme (Figure 9) that considers the normal and SCD signal classes. Table 2 shows the results of one of the tests and its metrics. The results showed that, in general, the evaluation criterion (Equation (3)) could identify a higher similitude between the input signal and its corresponding class, with an increased number of correct predictions. The accuracy (Acc) indicates that the correct classification of pre-SCD signals was higher than 90%. A general evaluation considering ten tests was performed to ensure the consistency of the results. Table 3 shows the statistics of the ten tests, where a high accuracy and low dispersion were observed at each time interval. Nevertheless, in the common scheme comparison for the pre-SCD intervals, it is likely that a signal differing from the normal class would be detected as SCD without considering the degree of difference, i.e., lower in further pre-SCD intervals and higher in the nearest pre-SCD intervals. This condition may cause the precision to have slight variations, despite the changing time interval.

A comparison with previous reports that performed pre-SCD signal classification under the common scheme using the MIT/BIH NSR and MIT/BIH SCDH databases is presented in Table 4. Data on the type of signal processed, methods, classifiers, and the prediction time, along with its respective accuracy, were also included. The comparison between these approaches and the proposed approach highlights the fact that the ECG signal is directly processed. Other methodologies used the HRV signal, but this increases the computational time, and a correction is required in the detection of R-R intervals [7]. Moreover, feature ranking is a common task in other works. Still, it is a complicated process, as the behavior of some features may change over time, meaning one feature evaluation per minute is needed to identify which features better represent that specific interval [19]. Since sparse representations provide a simplified description of the signal, α can be used as feature vector, avoiding feature ranking. Additionally, it was found that the normal and SCD signals can be identified with high precision using a simple criterion instead of a more sophisticated classifier. Acharya et al. [19] also proved a simple evaluation by using the Sudden Cardiac Index (SCDI) to detect SCD up to 4 min before the onset. In previous works, it was proven that it is possible to reach an SCD detection up to 30 min before onset, with a high accuracy.

**Figure 10:**
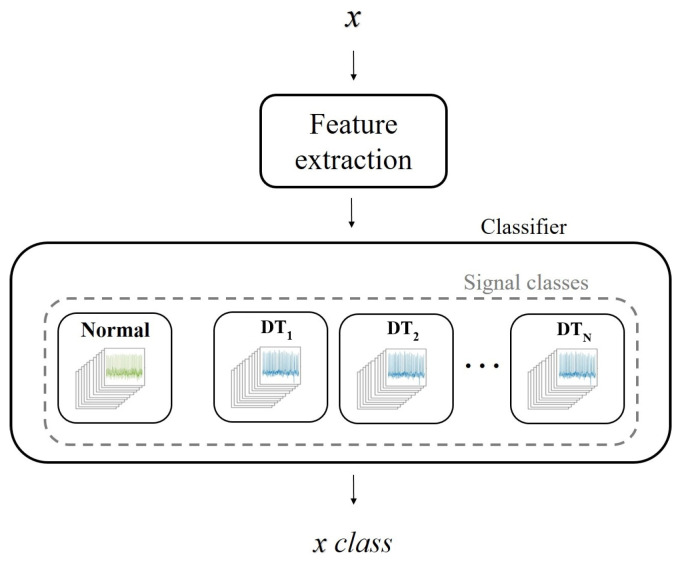
Proposed multi-class scheme for SCD ECG signal classification, in which is considered that differences with respect to normal signal do not necessarily correspond to an immediate SCD but to pre-SCD intervals or even other specific causes; the number of classes (*N*) depends on the conditions addressed in the experiment.

Although the traditional scheme (Figure 9) allows for comparison with the state-of-the-art SCD prediction, it might not be suitable to compare only two classes: normal signals and SCD signals. These SCD signals belong to patients with a history of heart disease [24]. Thus, the entire signal may behave differently than a normal signal, not just the signal in the minutes before an SCD event. For this reason, an experimental evaluation based on multiple classes was performed (Figure 10). In this case, the classes were associated with the time intervals defined in *C*. Since the features of the ECG signal change as the SCD gets closer, it is assumed that, by using different categories, local features (related with the proximity of SCD) could be highlighted, while common features (related to previous heart diseases) could be attenuated. In this way, the classification could be made more suitable.

Tests results under the proposed scheme are presented in Table 5; a two-fold cross-validation was computed, and repeated ten times. Since a class of normal signals was included, an approximation of the general results can be cmade, using the common scheme evaluated in Table 3. Unlike previous studies using ECG signals [17,18], it can be seen that the greater the distance from the start of an SCD event, the more difficult it is to predict the SCD with high accuracy. From the ten tests performed at the time intervals in *C*, an average accuracy of 80.5% was obtained for an SCD event up to 30 min in advance. The purpose of the experimental evaluation is the comparison of SCD signals with the same conditions of a history of heart disease; therefore, this is an evaluation with more equal conditions.

## 4. Conclusions

The early anticipation of SCD is vital to medical specialists who can apply preventive treatment, increasing survival. It was shown that dictionary learning is suitable to address ECG signals’ feature identification, and sparse representations are helpful as feature vectors. Moreover, since the ECG signal is sparse, through selecting the elemental signals that better represent it, feature ranking was not necessary under this approach. Additionally, because signal characterization was not based on fixed features but on elemental signals, it was possible to perform a feature extraction adapted to the dynamic of the ECG signals. The experiment performed under the common scheme showed that the methodology reached an accuracy similar or higher than related works, but by considering a wider pre-SCD interval. However, the binary evaluation under this scheme could be limited and bias the classification of pre-SCD signals. The proposed multi-class scheme was able to address the differences that were present among the pre-SCD signals, providing a more suitable classification. Furthermore, by considering that the features of the SCD attenuate as the SCD event gets closer, it was expected that identification of pre-SCD signals was reduced in longer intervals and increased in shorter intervals. This behavior corresponds with the results obtained under the multi-class scheme that reached an accuracy of 80.5% up to 30 min before SCD.

In future work, an analysis of elemental signals in dictionaries will be addressed to identify and filter those that generate noise in the α vector and affect signal classification, as was the case for the 5 min interval in Table 5. Since the aim is to detect an SCD episode in advance, we will seek to implement this methodology as embedded system for continuous monitoring.

## Figures and Tables

**Table 1:** Demographic information from the MIT/BIH-NSR and MIT/BIH-SCDH databases [23,24].

		Gender			Age	
	**Total**	**Male**	**Female**	**Unknown**	**Range**	**Mean**
SCD	23	13	8	2	17–82	60.31
Normal	18	5	13	-	20–50	34.33

**Table 2:** Results of an individual test under the common schemme.

Time Interval (before SCD)	*TP*	*TN*	*FP*	*FN*	*Acc* (%)
5-min	382	258	55	1	92.0
10-min	375	290	62	5	90.8
15-min	369	271	68	1	90.3
20-min	373	332	64	4	91.2
25-min	383	281	54	2	92.2
30-min	397	331	40	0	94.8

**Table 3:** Measures for the ten tests of ECG SCD classification through sparse representations.

Time Interval (before SCD)	*Acc* (%) ± std. dev.
5-min	94.4 ± 2.8
10-min	93.5 ± 2.7
15-min	92.7 ± 3.1
20-min	94.0 ± 3.1
25-min	93.2 ± 3.5
30-min	95.3 ± 2.5

**Table 4:** Prediction and accuracy comparison. The MIT/BIH NSR and MIT/BIH SCDH databases were used in all cases.

Work	Signal	Methods and Characterization	Classifier	Prediction Time (*Acc*)
U. Rajendra Acharya et al. (2015) [19]	HRV	Non-linear features extracted from DWT	k-NN	4 min before (86.8%)
U. Rajendra Acharya et al. (2015) [16]	ECG	Non-linear features extracted from DWT	SVM	4 min before (92.1%)
M. Murugappan et al. (2015) [21]	HRV	Time domain features	Fuzzy	5 min before (93.7%)
Hamido Fujita et al. (2016) [20]	HRV	Non-linear features extracted from DWT	SVM	4 min before (94.7%)
Elias Ebrahimzadeh et al. (2018) [22]	HRV	Non-linear, time-frequency, and linear features	MLP	12 min before (88.2%)
Amezquita-Sanchez et al. (2018) [18]	ECG	Non-linear feature from WPT	EPNN	20 min before (95.8%)
Olivia Vargas-Lopez et al. (2020) [17]	ECG	Non-linear features from EDM	MLP	25 min before (94%)
Proposed	ECG	Sparse Representations	Sum of absolute α	30 min before (95.3%)

**Table 5:** ECG SCD classification through sparse representations based on the proposed multi-class scheme.

Time Interval (before SCD)	Accuracy (%) ± std. dev.
Normal minute	96.3 ± 1.4
5-min	86.2 ± 0.9
10-min	78.4 ± 1.6
15-min	80.1 ± 2.1
20-min	81.0 ± 1.3
25-min	83.8 ± 1.0
30-min	80.5 ± 2.8
